# Effect of Strain Rate on Compressive Behavior of a Zr-Based Metallic Glass under a Wide Range of Strain Rates

**DOI:** 10.3390/ma13122861

**Published:** 2020-06-25

**Authors:** Wenqing Li, Tieqiang Geng, Shaofan Ge, Zhengwang Zhu, Long Zhang, Zhengkun Li, Huameng Fu, Hongwei Zhang, Hong Li, Aimin Wang, Haifeng Zhang

**Affiliations:** 1 School of Materials Science and Engineering, University of Science and Technology of China, Hefei 230026, China; wqli13s@imr.ac.cn (W.L.); sfge16b@imr.ac.cn (S.G.); 2 Institute of Metal Research, Shenyang National Laboratory for Materials Science, Chinese Academy of Sciences, Shenyang 110016, China; tqgeng@imr.ac.cn (T.G.); zhanglong@imr.ac.cn (L.Z.); zkli@imr.ac.cn (Z.L.); hmfu@imr.ac.cn (H.F.); hongweizhang@imr.ac.cn (H.Z.); lihong@imr.ac.cn (H.L.); amwang@imr.ac.cn (A.W.)

**Keywords:** amorphous alloy, strain rate, yield strength, free volume, thermal softening, SHPB

## Abstract

The strain rate effect on the mechanical behavior of amorphous alloys has aroused general interest. Most studies in this area have focused on quasi-static and high strain-rate compressive deformations. However, experimental results have been few, or even non-existent, under a moderate strain-rate loading. This article extends the traditional split Hopkinson pressure bar (SHPB) technique to characterize compressive deformation of an amorphous alloy at medium strain rates. The compressive behavior of Zr_65.25_Cu_21.75_Al_8_Ni_4_Nb_1_ amorphous alloy shows a negative strain rate effect on the yield strength with a quasi-static, moderate to high strain-rate range, and the fracture angle increases from 44° at 10^−5^ s^−1^ to 60° at 4000 s^−1^ as strain rate increases_._ Herein, we introduce a modified cooperative shear model to describe the compressive behavior of the current amorphous alloy under a broad strain rate range. The model predicts that the normalized yield strength will linearly descend with logarithmic strain rate when the strain rate is less than a critical strain rate, however, which rapidly decreases linearly with the square of the strain rate at high strain rates. The predicted data of the model are highly consistent with the current experimental results. These findings provide support for future engineering applications of amorphous alloys.

## 1. Introduction

Amorphous alloys have long been studied in terms of their high strength, high hardness, and high fracture toughness [1,2,3]. The deformation behavior under different strain rates has been widely regarded. Since Bruck et al. [4] studied the high strain rate deformation behavior of Vit1 amorphous alloy (Zr_41.25_Ti_13.75_Cu_12.5_Ni_10_Be_22.5_) using a split Hopkinson pressure bar (SHPB) test technique, numerous researchers have also studied a large number of strain rate effects in other amorphous systems [5,6,7,8,9,10,11,12,13,14,15,16,17], although a consensus is far from being reached. For example, for Vit1 alloy, Sunny et al. [10] and Bruck et al. [4] believed that the strain rate effect on the strength is slight, and Hufnagel et al. [5] reputed the idea of a negative strain rate effect. For most other types of amorphous alloy, there is a negative strain rate effect [18,19,20].

Owing to the brittleness of amorphous alloys, the compression behavior under different strain rates has received the most focus. The quasi-static range (a strain rate of usually smaller than 0.1 s^−1^) is characterized using a universal testing machine. A high strain rate range (10^3^–10^4^ s^−1^) is usually characterized using SHPB technology. Higher strain rates (generally greater than 10^4^ s^−1^) are characterized by light gas guns and flying fin tests (however, they usually have a complex state of stress, which is difficult to match with a uniaxial state of stress). According to the general principle applied to a brittle material test of an SHPB, compressive loading cannot be achieved at a constant strain rate of above 10^4^ s^−1^ [21]. For amorphous alloys, however, few experimental results have been obtained within a strain rate range of 10^2^–10^3^ s^−1^, and there are no test data available within the medium strain rate range of 1–10^2^ s^−1^. This is mainly because a universal testing machine cannot reach such a high loading speed and a traditional SHPB cannot achieve such a low strain rate owing to the limitation of the loading time. It is apparent that the shortage of the mechanical parameters obtained at medium strain rates largely influences the verification of the mechanical theories and models, thereby hindering the in-depth understanding, and style of high-performance amorphous alloys.

Over the past few decades, the free volume model [22,23] and cooperative shear model (CSM) [24] are mostly applied toward the prediction of the deformation behaviors of amorphous alloys at different strain rates. In particular, after the modification by Liu et al. [12], the CSM model has shown good agreement with the previously reported experimental results. However, the model predicts the occurrence of a slight positive strain rate on the yield strength within the range of medium strain rates, which has yet to be verified. To overcome the drawback of using an extremely long striker bar, which is necessary for conventional medium strain rate loading, a new method obtaining a long duration based on a pulse shaper technique with a short striker is introduced herein. In addition, a set of SHPB testing techniques developed by Zhao et al. [25], which have an advantage of no recording time limit and were first applied to characterize the mechanical behaviors of amorphous alloys under medium strain rates, are described. Based on these techniques, a new method for characterizing the deformation of amorphous alloys at moderate strain rates was developed. In addition, the deformation behaviors of Zr_65.25_Cu_21.75_Al_8_Ni_4_Nb_1_ amorphous alloys under quasi-static, medium strain rates, and high strain rates were studied. Based on the latest experimental results, we improved the CSM model, and achieved consistency over a wide range of strain rates.

## 2. Materials and Methods

A master alloy with a nominal composition of Zr_65.25_Cu_21.75_Al_8_Ni_4_Nb_1_ (in atomic percentage, at %, denoted as ZC3) was prepared by arc-melting mixtures of the element metals under a Ti-gettered argon atmosphere. The element metal has a purity of as high as 99.9%. The chemical homogeneity was obtained by re-melting the metal a minimum of four times. As-cast samples with a diameter of 4 mm and a length of 60 mm were prepared using a copper-mold tilting casting method. The microstructures of the samples were characterized using X-ray diffraction (XRD; Rigaku D/max 2400, Japan, Cu-Kα). Thermal properties concerning the glass transition and crystallization were examined using differential scanning calorimetry (DSC; Netzsch 204F) in a flowing argon atmosphere at a heating rate of 20 K/min. The as-cast and deformed samples were observed using a scanning electron microscope (SEM, FEI Quanta 600). The specimens with a size of 4 mm (diameter) × 4 mm (length) used for mechanical tests were cut from the as-cast samples. All specimens were mechanically polished using No. 2000 SiC paper with the ends carefully ensured to be in a parallel position. Quasi-static compression tests were conducted on an Electronic Universal Testing Machine (Instron 5582) with strain rates of 10^−5^, 10^−4^, 10^−3^, 10^−2^, and 10^−1^ s^−1^. High strain rate experiments were conducted on a conventional SHPB with strain rates of 300, 1500, 2500, 4400, and 5600 s^−1^, more details of which can be found in [4] and [26]. As the main change, the position of the strain gage was closer to the left end instead of in the middle of the incident and transmission bars, which is an effective method for increasing measurement duration of an SHPB system [27,28], and thus we could employ a compression test at a strain rate of 200–1000 s^−1^. In addition, to achieve strain rates of 50–100 s^−1^, the SHPB technique was further explored.

For a typical SHPB experiment with a 300-mm length striker bar and a thin pulse-shaper on amorphous glass with a fracture strain $\varepsilon_{f}$, the effective loading time is $t_{0}$; in addition, the strain rate can be estimated using $\varepsilon_{f}/t_{0}$, $t_{0}$ = 5–120 μs, $\varepsilon_{f}$ = 2–3%, at which the strain rate is approximately 300–6000 s^−1^. For a classic SHPB, to separate the reflected and incident waves within the same strain gauge, the length of the striker bar is limited to half the length of the incident bar, and $t_{0}$ is proportional to the length of the striker bar. To obtain a lower strain rate, $t_{0}$ must be increased. This means that the lengths of the striker, incident and transmission bars must be increased proportionally. If the strain rate is 100 s^−1^ and $t_{0}$ is 300 ms, we need a 1.5-m-long striker bar, a 3-m-long incident bar, and a transmission bar. To obtain a lower strain rate, the SHPB test system will be larger, which will be costly and inefficient.

To achieve lower strain rates without increasing the length of the striker, incident, and transmission bars, two issues need to be addressed. One is the use of specially designed shapers and a short striker bar to achieve a long-duration incident wave, and the other is an effective method of separation of the stress waves, namely, a two-point measurement, as proposed by Zhao et al. [25]. Using the two-point measurement technique [25,29], we use a 0.4-m long striker bar and a suitable shaper to achieve an effective loading time in excess of 1 m s, the strain rate of which can reach approximately 20 s^−1^.

As is well known, a pulse shaper that changes the profile of the incident wave can effectively increase the wave duration. However, the duration of the incident wave with a common pulse-shaper generally does not exceed 50% without a pulse shaper. Initially, a pulse shaper can significantly increase the duration of the incident wave with a short striker bar. For instance, for a 400-mm long striker, the duration of the incident wave is approximately 200 μs using a common pulse shaper, although we can obtain the 600–700 μs duration of an incident wave using a special copper pulse shaper. Furthermore, the constant strain rate loading is extremely important for the SHPB test. We change the striker bar velocity, striker length, diameter and thickness pulse shaper to obtain a linear incident wave which is considered an ideal incident wave in which a brittle material deforms under a constant strain rate loading when using an SHPB [30].

**Figure 1:**
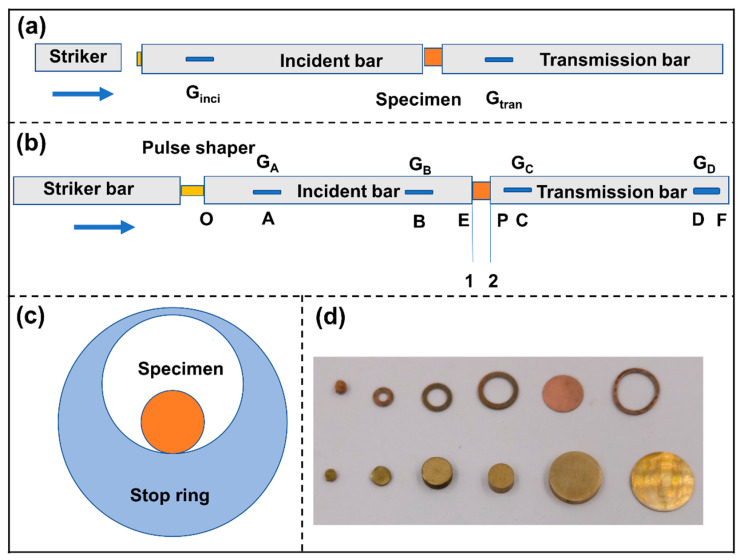
Schematic diagram of conventional and modified split Hopkinson pressure bar (SHPB). (**a**) conventional SHPB; (**b**) modified SHPB for moderate strain rate loading; (**c**) specimen assembly and (**d**) brass and copper pulse shapers with different shapes and sizes.

A schematic profile of a classic SHPB is shown in Figure 1a. The basic principle of an SHPB is the use of the strain history of incident and transmission bar strain gages to obtain the strain rate and stress history of the specimen, as shown in Equations (1)–(3) [26]:

(1) $${\dot{\varepsilon }}_{s}\left(t\right)=\frac{c_{0}}{l_{s}}\left(\varepsilon_{i}\left(t\right)-\varepsilon_{r}\left(t\right)-\varepsilon_{t}\left(t\right)\right),$$

(2) $$\varepsilon_{s}\left(t\right)=\frac{c_{0}}{l_{s}}\int_{0}^{t}\left(\varepsilon_{i}\left(\tau \right)-\varepsilon_{r}\left(\tau \right)-\varepsilon_{t}\left(\tau \right)\right)d\tau ,$$

(3) $$\sigma_{s}\left(t\right)=\frac{A_{0}E_{0}}{2A_{s}}\left(\varepsilon_{i}\left(t\right)+\varepsilon_{r}\left(t\right)+\varepsilon_{t}\left(t\right)\right),$$

the incident wave $\varepsilon_{i}\left(t\right)$, reflected wave $\varepsilon_{r}\left(t\right)$, and transmitted wave $\varepsilon_{t}\left(t\right)$ are subsequently separated from the filtered strain signal. In addition, ${\dot{\varepsilon }}_{s}\left(t\right)$, $\varepsilon_{s}\left(t\right)$ and $\sigma_{s}\left(t\right)$ are the strain rate history, strain history, and stress history of the specimen; $c_{0}$ is the wave velocity of an incident and transmission bar; $A_{s}$ and $A_{0}$ are the cross-sectional area of a specimen and bar, respectively; $l_{s}$ is the specimen thickness; and $E_{0}$ is the Young’s modulus of the bar. 

For a valid SHPB test, the stress of the interface between the bar and specimen is equal during loading and unloading, which means

(4) $$\sigma_{i-s}\left(t\right)=\sigma_{t-s}\left(t\right),$$

where $\sigma_{i-s}\left(t\right)$ and $\sigma_{t-s}\left(t\right)$ are the incident and transmission interface stresses denoted by i−s and t−s, respectively (interface between the sample and bars).

For the more general principle according to the elastic stress wave theory, the strain ε(t) at each section is the sum of the contribution of the elementary right-going wave $\varepsilon_{R}\left(t\right)$ and elementary left-going wave $\varepsilon_{L}\left(t\right)$, and the velocity v(t) is proportional to their difference [25]. For a modified SHPB, as shown in Figure 1b, if we can obtain the strain and velocity history of the end of the incident bar $\varepsilon_{1}\left(t\right),\;v_{1}\left(t\right)$ and the start of the transmission bar $\varepsilon_{2}\left(t\right),\;v_{2}\left(t\right)$, i.e.,

(5) $$\varepsilon_{1}\left(t\right)=\varepsilon_{R1}\left(t\right)+\varepsilon_{L1}\left(t\right),$$

(6) $$v_{1}\left(t\right)=c_{0}(\varepsilon_{R1}\left(t\right)-\varepsilon_{L1}\left(t\right)),$$

(7) $$\varepsilon_{2}\left(t\right)=\varepsilon_{R2}\left(t\right)+\varepsilon_{L2}\left(t\right),$$

(8) $$v_{2}\left(t\right)=c_{0}(\varepsilon_{R2}\left(t\right)-\varepsilon_{L2}\left(t\right)),$$

We can compute the strain rate, strain and stress history of the specimen as follows [29]:

(9) $${\dot{\varepsilon }}_{s}\left(t\right)=\frac{v_{2}\left(t\right)-v_{1}\left(t\right)}{l_{s}},$$

(10) $$\varepsilon_{s}\left(t\right)=\int_{0}^{t}\frac{v_{2}\left(\tau \right)-v_{1}\left(\tau \right)}{l_{s}}d\tau ,$$

(11) $$\sigma_{s}\left(t\right)=\frac{A_{0}E_{0}}{2A_{s}}\left(\varepsilon_{1}\left(t\right)+\varepsilon_{2}\left(t\right)\right),$$

If we obtain the elementary right-going wave $\varepsilon_{R1}\left(t\right)$, $\varepsilon_{R2}\left(t\right)$ and elementary left-going wave $\varepsilon_{L1}\left(t\right)$, $\varepsilon_{L2}\left(t\right)$ at the end of incident bar and at the start of the transmission bar, we can easily obtain the strain and stress histories. The key is to obtain the two elementary waves according to the strain history of the incident strain gages and the transmission strain gages. The details of this process are described in the following.

For completeness, the right-going and left-going waves on the incident bar with the strain history of sections A and B are calculated explicitly as follows [31]:

(12) $$\{\begin{array}{c} \varepsilon_{RA}\left(t\right)=\varepsilon_{A}\left(t\right),t\leq T_{A}\\ \varepsilon_{RA}\left(t\right)=\varepsilon_{A}\left(t\right)-\varepsilon_{B}\left(t-T_{AB}\right)+\varepsilon_{RA}\left(t-2T_{AB}\right),t\geq T_{A} \end{array}’$$

(13) $$\{\begin{array}{c} \varepsilon_{LB}\left(t\right)=0,\;t\leq T_{B}\\ \varepsilon_{LB}\left(t\right)=\varepsilon_{B}\left(t\right)-\varepsilon_{A}\left(t-T_{AB}\right)+\varepsilon_{LB}\left(t-2T_{AB}\right),t\geq T_{B} \end{array}’$$

where *T_AB_* = *L_AB_*/*c_0_*,$\;T_{A}=\left[2\left(L_{AB}+L_{BE}\right)+L_{OA}\right]/c_{0}$, and $T_{B}=\left(2L_{BE}+L_{OA}+L_{AB}\right)/c_{0}$. For the interface between the incident bar and the specimen, the right-going and left-going waves can be calculated as follows [32]:

(14) $$\varepsilon_{R1}\left(t\right)=\varepsilon_{RA}\left(t-\frac{L_{AB}+L_{BE}}{c_{0}}\right),\;$$

(15) $$\varepsilon_{L1}\left(t\right)=\varepsilon_{LB}\left(t+\frac{L_{BE}}{c_{0}}\right){,}_{\;}$$

Similarly, the right-going and left-going waves on the transmission bar with the strain history of sections C and D are calculated as follows [31]:

(16) $$\{\begin{array}{c} \varepsilon_{RC}\left(t\right)=\varepsilon_{C}\left(t\right),t\leq T_{C}\\ \varepsilon_{RC}\left(t\right)=\varepsilon_{C}\left(t\right)-\varepsilon_{D}\left(t-T_{CD}\right)+\varepsilon_{RC}\left(t-2T_{CD}\right),t\geq T_{C} \end{array}’$$

(17) $$\{\begin{array}{c} \varepsilon_{LD}\left(t\right)=0,\;t\leq T_{D}\\ \varepsilon_{LD}\left(t\right)=\varepsilon_{D}\left(t\right)-\varepsilon_{C}\left(t-T_{CD}\right)+\varepsilon_{LD}\left(t-2T_{CD}\right),t\geq T_{D} \end{array}’$$

where $T_{C}=\left[2\left(L_{CD}+L_{DF}\right)+L_{PC}\right]/c_{0}$, $T_{D}=\left(2L_{DF}+L_{PC}+L_{CD}\right)/c_{0}$, and *T_CD_* =*L_CD_*/*c*_0_. For the interface between the transmission bar and the specimen, the right-going and left-going waves can be calculated as follows [32]:

(18) $$\varepsilon_{R2}\left(t\right)=\varepsilon_{RC}\left(t+\frac{L_{PC}}{c_{0}}\right),$$

(19) $$\varepsilon_{L2}\left(t\right)=\varepsilon_{LD}\left(t-\frac{L_{PC}+L_{CD}}{c_{0}}\right){,}_{\;}$$

Thus, we can go back to Equations (5)–(11) to obtain the strain rate history, strain history, and stress history of the specimen.

The modified SHPB and sample setup values are *c_0_* = 5000 m/s, *E_0_* = 210 GPa, *A_0_* = 165.13 mm^2^, *A_s_* = 12.56 mm^2^, *l_s_* = 4 mm, *L_OA_* = 215 mm, *L_AB_* = 590 mm, *L_BE_* = 195 mm, *L_PC_* = 205 mm, *L_CD_* = 588 mm, and *L_DF_* = 207 mm.

A unique limit ring is used to avoid continuous loading after an amorphous alloy fracture. Figure 1c shows a schematic of a sample and a stop ring assembly. Pulse shapers made by brass and copper of different diameters, thicknesses and shapes are shown in Figure 1d.

## 3. Results and Discussion

### 3.1. Medium and High Strain Rate Loading Using SHPB

#### 3.1.1. Incident Waves under Different Pulse Shapers

**Figure 2:**
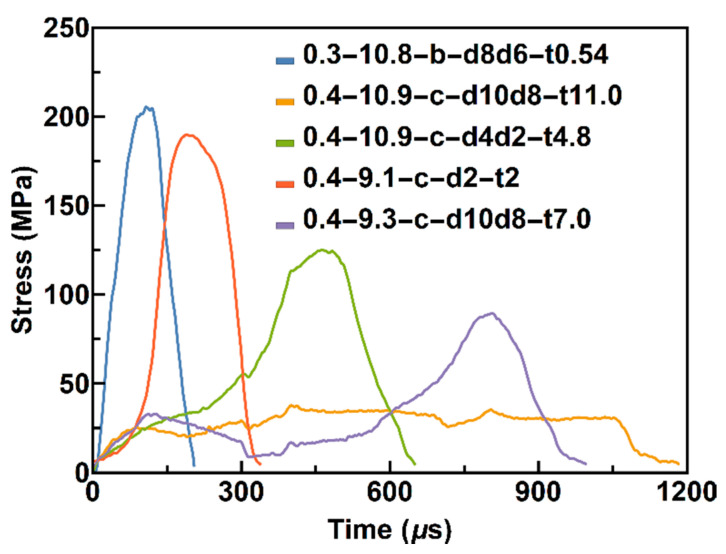
Incident waves using different pulse shapers.

Incident waves with different durations are necessary to obtain different constant strain rate loading levels when using pulse shapers. As shown in Figure 2, the striker bar is 300 and 400 mm (denoted by 0.3 and 0.4) long. The velocity of the striker bar is approximately 9–11 m/s (marked by the value after the length of the striker bar), the brass and copper pulse shapers are denoted by b and c, respectively, d4 indicates for the diameter of a 4 mm plate, d4d2 denotes an annular pulse shaper with an outer diameter of 4 mm, and an inner diameter of 2 mm, and t0.5 denotes a thickness of 0.5 mm. The duration will clearly increase if the L/D of the pulse shaper is greater than 1. As shown in Figure 2, an incident wave of approximately 1 m s will be achieved using a 400 mm long striker and a suitable pulse shaper, which provide the possibility of strain rates of less than 30 s^−1^.

#### 3.1.2. SHPB Curves under a Strain Rate of 300 s^−1^

**Figure 3:**
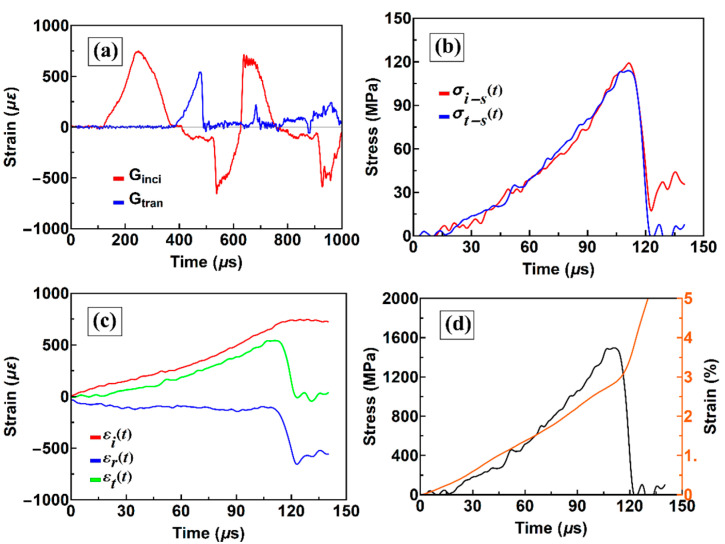
Typical signals of a conventional SHPB: (**a**) original stress signals from two incident and transmission gauges; (**b**) interface stress between the bar and sample; (**c**) incident, reflected, and transmission waves, and (**d**) stress and strain history curves of the sample.

For a classic SHPB, as shown in Figure 3a, the incident and reflected stress waves were separated on the original incident strain gauge signal. Figure 3b shows that the stress balance is obtained during the sample loading and unloading, which means that the SHPB test is valid. Figure 3c shows that the $\varepsilon_{r}\left(t\right)$ is almost a horizontal line, indicating that an almost constant strain rate is achieved during the test. According to the stress and strain history of the sample in Figure 3d, the strain rate is approximately 300 s^−^^1^, and the strength is 1500 MPa. In the experiment testing the amorphous compressive properties using the SHPB, due to the small size of the amorphous alloy, it is difficult to reach a strain rate of less than 500 s^−1^. Only Zhou et al. [16,17] have reached a strain rate of 300 s^−1^.

#### 3.1.3. SHPB Curves under a Strain Rate of 100 s^−1^

**Figure 4:**
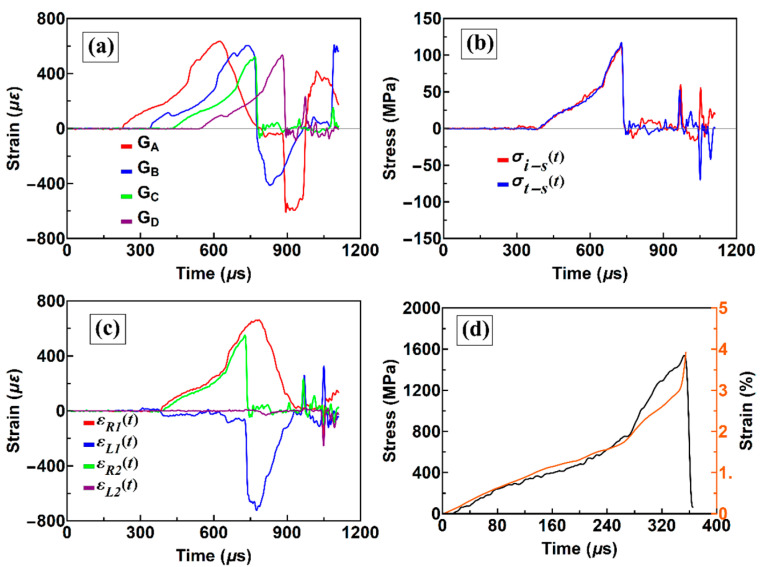
Typical signals of a modified SHPB: (**a**) original stress signals from four incident and transmission gauges; (**b**) interface stress between the bar and sample; (**c**) four right-going and left-going stress waves; and (**d**) the stress and strain history curves of the sample.

Figure 4a shows four strain gage signals. Figure 4b indicates that the equilibrium between both sides of the sample is nearly achieved during the loading and unloading. Figure 4c shows two waves spreading in the opposite directions on both the left and right sides. Among them, $\varepsilon_{L1}\left(t\right)$ represents the strain rate history and is extremely low, which suggests that a comparatively low strain rate is obtained. According to the stress and strain histories of the sample in Figure 4d, the strain rate is approximately 100 s^−1^ and the strength is 1520 MPa.

### 3.2. Stress-Strain Relationship at Different Strain Rates

**Figure 5:**
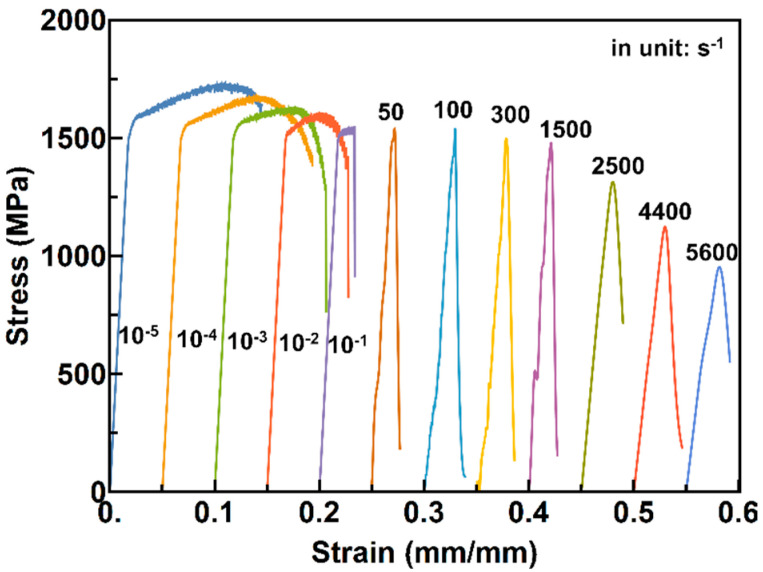
Stress–strain relationship of ZC3 under different strain rates.

Compression stress–strain curves at different strain rates are shown in Figure 5. It can be seen from the figure that as the strain rate increases, the fracture strength gradually decreases from 1700 to 1000 MPa, and the plasticity decreases from 5% at 10^−5^ s^−1^ to 0.5% at 0.1 s^−1^. Plasticity is almost zero at higher strain rates. Being deformed at moderate and high strain rates, the fracture strength remains almost the same from 50 to 300 s^−1^ and then decreases rapidly from 1460 MPa at 1500 s^−1^ to 1110 MPa at 5600 s^−1^. The plasticity remains at zero when the strain rate is greater than 50 s^−1^.

### 3.3. Fracture Morphology at Different Strain Rates

**Figure 6:**
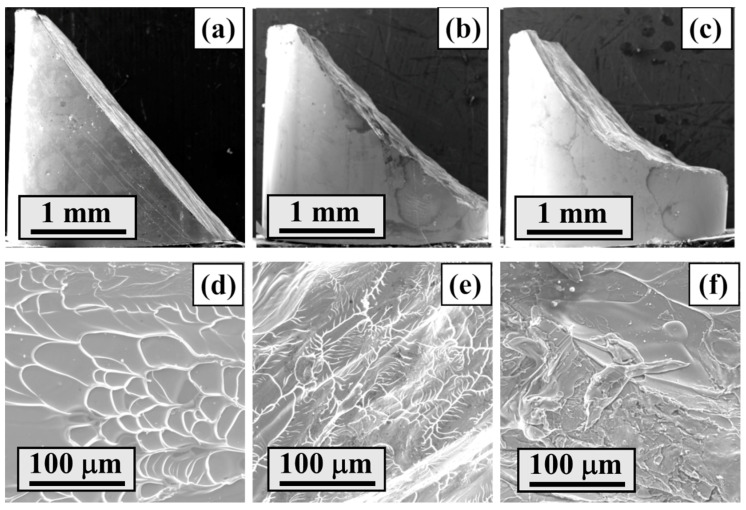
Side view and fracture surface details of ZC3 under different strain rates (**a**,**d**) 10^−5^ s^−1^, (**b**,**e**) 100 s^−1^, and (**c**,**f**) 2500 s^−1^.

To avoid destroying the initial fracture morphology, a special limit deformation ring is added [33]. The side morphology of the sample after compression deformation at different strain rates is shown in Figure 6a–c. It can be seen that the fracture angle differs at different strain rates, which is consistent with the results of Zhou et al. [16] and Fan et al. [13].

As indicated by Fan [15], unlike quasi-static deformation, in the shear bands under a high strain rate loading, the temperature rise and resultant melting phenomenon are expected to be dependent on the strain rate, the melting phenomenon seems to reach a serious level, and the width of the vein-like structure increases with an increase in the strain rate, as shown in Figure 6d–f.

**Figure 7:**
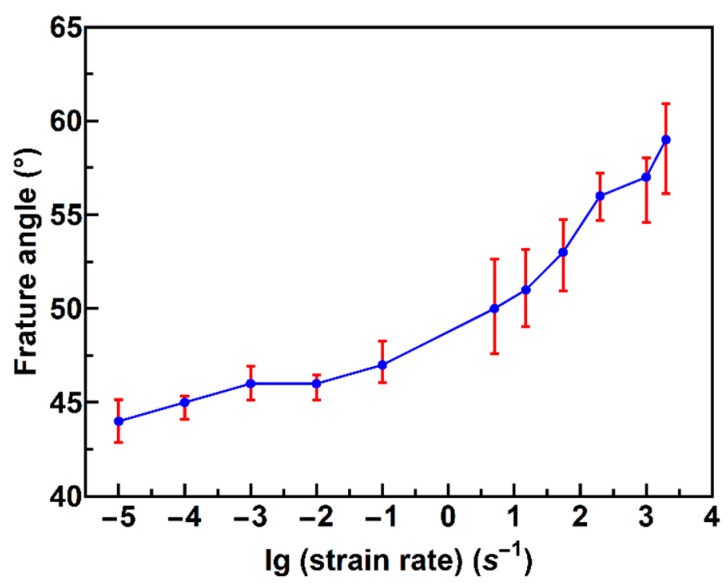
Variation of fracture angle based on the strain rate.

We calculated the fracture angle, using photo-processing software. Because the fracture surface does not remain flat under intermediate and high strain rates, we take a medium value of the fracture angle as the fracture angle. As shown in Figure 7, the fracture angle increases with an increase in the strain rate.

### 3.4. Model Prediction of Yield Strength at Different Strain Rates

It is widely accepted that the shear transformation zone (STZ) is the embryo of the localized shear event in amorphous alloys at the atomic scale, and the yield of amorphous alloys corresponds to the unsteady propagation of a large number of STZs [22,23]. The cooperative shear model of the STZs developed by Johnson and Samwer [24] gave a universal law for the shear yield strength of amorphous alloys as follows:

(20) $$\tau_{T}=\tau_{0}-\tau_{0}{\left[\left(\frac{k}{\beta }\right)ln\frac{\omega_{0}}{C\dot{\gamma }}\left(\frac{G_{0T}}{G_{0T_{g}}}\right)\right]}^{\frac{2}{3}}{\left(\frac{T}{T_{g}}\right)}^{\frac{2}{3}},\;$$

where $\tau_{T}$ and $\tau_{0}\;$ are the shear yield strength at a finite temperature *T* and absolute zero temperature, respectively; C and β are constants, and $\dot{\gamma }$ is the shear strain rate. $T_{g}$ is the glass transition temperature; k and $\omega_{0}$ are the Boltzmann constant and attempted frequency, respectively; and $G_{0T}/G_{0T_{g}}$ is the ratio of the shear modulus at finite temperature T and $T_{g}$, incorporating the weak dependence of the shear modulus on the thermal expansion of a fixed glass configuration. Liu [12] deduced an equation of the temperature increase around an emerging STZ as

(21) $$T=T_{r}+\frac{\xi \Omega \eta {\dot{\gamma }}^{2}}{8\pi \kappa r_{0}},\;$$

where *T_r_* is room temperature, ξ is a correction factor, and Ω is the STZ volume. In addition, η and κ are the viscosity and thermal conductivity near the contiguous STZ, respectively; $r_{0}$ is the characteristic radial distance of the heat source, which should be approximately equal to the radius of the STZ; and $\tau_{r}$ and $\tau_{0}$ are the quasi-static shear yield strength at room and absolute zero temperatures, respectively, with the following relationship [24]:

(22) $$\tau_{0}=\tau_{r}/\left[\;1-{\left(0.2T_{r}/T_{g}\right)}^{2/3}\right],\;$$

According to the stress and strain transformation relationships in elastic mechanics, the shear and normal stresses on the fracture plane can be derived from:

(23) $$\tau_{\theta }=\sigma_{f}sin\theta cos\theta ,\;$$

(24) $$\gamma_{\theta }=2\varepsilon_{f}sin\theta cos\theta ,\;$$

where $\tau_{\theta }$ and $\gamma_{\theta }$ are the shear stress and strain on the fracture plane, and $\sigma_{f}$ and $\varepsilon_{f}$ are fracture stress and strain, respectively. Therefore, the uniaxial yield strength $\sigma_{y}$ can be derived from the following:

(25) $$\sigma_{y}=\tau_{y}/sin\theta cos\theta ,$$

where $\tau_{y}$ is the shear yield strength. When Equation (24) is divided by the effective loading time, we can derive the average shear strain rate $\dot{\gamma }$ as follows:

(26) $$\dot{\gamma }=2\dot{\varepsilon }sin\theta cos\theta ,$$

where $\dot{\varepsilon }$ is the average uniaxial compressive strain rate. 

As shown in Figure 7, the fracture angle is changed with the strain rate, unlike in the approaches by Liu [12] and Li [14], who considered it to be a constant.

(27) $$\theta =\theta \left(\dot{\varepsilon }\right),$$

In deriving the Equation (20) [24], the most important information was the potential energy barrier as a function of the shear stress and temperature according to the scale law. By contrast, the relationship between the barrier and shear strain rate obeys the classic Arrhenius temperature equation. In [24], considering that the shear strain rate is beyond a narrow range (~10^−3^ s^−1^), an order of magnitude change in either $\omega_{0}$ or $\dot{\gamma }$ changes the logarithmic term by ~5%, and it is reasonable for the approach in [24] to treat $\omega_{0}$ and C as a constant for different alloys. Li et al. [14] and Liu et al. [12] both argued that $\omega_{0}$ and C are constant when the shear strain rate is changing rapidly, which resulted in their models showing an apparent positive strain rate effect when the strain rate is less than 10^3^ s^−1^. Based on present study results, we believe that C is a function of shear strain rate $\dot{\gamma }$ and composition. Two parameters are introduced:

(28) $$C=C_{0}{\left(\dot{\gamma }/\dot{\gamma_{0}}\right)}^{\alpha_{1}},\;\alpha_{2}=\ln\left(\omega_{0}/C_{0}\right),$$

where $\alpha_{1}$ stands for an alloy composition effect and $\dot{\gamma_{0}}$ is a reference shear strain rate, where $\dot{\gamma_{0}}$ = 10^−5^ s^−1^. In addition, $C_{0}$ is a dimensionless constant of order unity, and $\alpha_{2}$ is an assistant parameter for simplifying $\omega_{0}$ and $C_{0}$. Otherwise, for further simplification, three constants are introduced:

(29) $$\zeta_{0}=1/\left(1-{\left(0.2T_{r}/T_{g}\right)}^{2/3}\right),\alpha_{0}=\left(\kappa /\beta \right)G_{0T}/G_{0T_{g}},\beta_{0}=\xi \Omega \eta /8\pi \kappa r_{0},$$

Substituting Equations (25), (28) and (29) into Equation (20): the following is derived:

(30) $$\frac{\sigma_{y}}{\sigma_{r}}=\frac{Sin\left(2\theta_{0}\right)}{Sin\left(2\theta \right)}\left\{\zeta_{0}-\zeta_{0}{\left[\alpha_{0}\left(\alpha_{2}-ln\left(\dot{\gamma }{\left(\frac{\dot{\gamma }}{\dot{\gamma_{0}}}\right)}^{-\alpha_{1}}\right)\right)\right]}^{\frac{2}{3}}{\left(\frac{T_{r}+\beta_{0}\dot{\gamma^{2}}}{T_{g}}\right)}^{2/3}\right\},$$

By substituting Equations (26) and (27), we can conclude that $\dot{\gamma }=\dot{\varepsilon }sin\left(2\theta \left(\dot{\varepsilon }\right)\right)$,$\sigma_{r}=2\tau_{r}/Sin2\theta_{0}$ is the quasi-static yield strength at room temperature, and $\theta_{0}$ is the quasi-static fracture angle at room temperature. According to [12], for ZC3 amorphous alloy, these constants can take values of $T_{r}$ = 293 K, $T_{g}$ = 654 K, $\zeta_{0}$ = 1.25, $\alpha_{0}$ = 0.0057 and $\beta_{0}$ = 2.2 × 10^−5^ K·s^2^. Considering that some basic parameters cannot be determined precisely, simply taking the parameters in [12,14,24] as the initial guess values of $\alpha_{1}$ and $\alpha_{2}$, and then using the Levenberg–Marquardt Method to solve the nonlinear fitting, we obtain that:${\;\alpha }_{1}$ = 1.3, $\alpha_{2}$ = 24.5.

**Figure 8:**
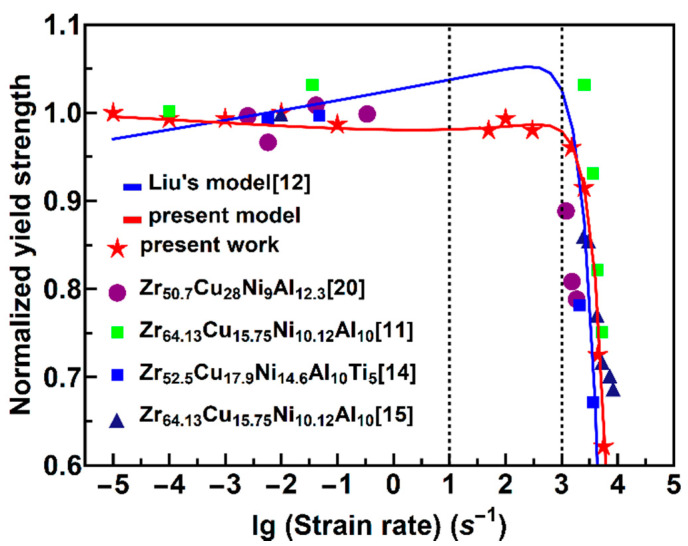
Relationship between the normalized yield strength and the strain rate.

By substituting all of the parameters into Equation (30), we can obtain the relationship between the normalized yield strength $\sigma_{y}/\sigma_{r}$ and the applied average strain rate $\dot{\varepsilon }$ at room temperature. Liu’s [12] model and the current model are shown in Figure 8, which are represented by black and purple lines, respectively. Comparing the two lines, we can see that the critical strain rate, when considering the changes in the fracture angle with strain rate, is larger than the constant fracture angle. The fracture angles of Zr_52.5_Cu_17.9_Ni_14.6_Al_10_Ti_5_ [14] and Zr_50.7_Cu_28_Ni_9_Al_12.3_ [20] are considered to be 43° and 42°–43°, respectively, and remain constant with the strain rate. Therefore, their results are more consistent with Liu’s model [12]. However, the results of the present study and Zr_55_ Cu_30_Al_10_Ni_5_ [13,18] indicate that the fracture angle of the two alloys increases with an increase in the strain rate. In addition, Zr_64.13_Cu_15.75_Ni_10.12_Al_10_ [11] showed a small part of fractured sample with an inconsistent fracture angle, which means the fracture surface of the bigger part is similar to that of Zr_55_Cu_30_Al_10_Ni_5_ [13]. In other words, for those amorphous alloys in which the fracture angle changes with the strain rate, the current model is more consistent with the experimental results of present work and Zr_64.13_Cu_15.75_Ni_10.12_Al_10_ [11,15], as shown in Figure 8.

The model indicates that the yield strength remains constant when the strain rate is not exceeded by the critical strain rate. For the present amorphous alloy, the critical strain rate is approximately 800–1000 s^−1^, and the yield strength decreases severely when the strain rate exceeds the critical strain rate. Because the range of 10^3^–10^4^ s^−1^ is extremely narrow, the yield strength decreases rapidly with the strain rate.

In the following, analyzing the Equation (30), when the strain rate is less than 1 s^−1^, the temperature rise in Equation (21) is extremely small. This formula can be simplified into a linear relationship: $\frac{\sigma_{y}}{\sigma_{r}}\propto -lg\dot{\varepsilon }$, although the slope is extremely small, and the yield strength decreases slowly with an increase in the strain rate. By contrast, when the strain rate is greater than 10^3^ s^−1^, the temperature rise is extremely significant, and the left part remains mostly constant. Thus, the formula in Equation (30) can be simplified as a square relationship, $\frac{\sigma_{y}}{\sigma_{r}}\propto -{\dot{\varepsilon }}^{2}$. Therefore, at a high strain rate, the yield strength decreases rapidly with an increase in the strain rate. For a compressive yield strength under quasi-static, medium, and high strain rates, the model is quite consistent with the experimental results. 

We also attempted to understand the underlying mechanism. In an amorphous alloy specimen experiencing a uniform stress, it will be easier for the activation of STZ to occur in local regions with a higher free volume concentration [22,23], indicating a free volume softening effect on the yield strength. At the same time, it will be more difficult for the STZs to occur owing to a smaller available free volume under a higher strain rate, producing a strain rate hardening effect on the yield strength. A higher temperature will facilitate a quick activation and combination of the STZs, which will result in a rapid formation of a shear band. Consequently, it is reasonable to conclude that a free volume softening, a strain rate hardening, and a thermal softening occur under different strain rates [14]. When the strain rate increases to greater than a critical value, the combination of these three factors eventually results in a significant negative dependence of the yield strength on the strain rate. However, the strain rate effect on the yield strength becomes complex at a moderate strain rate, which is generally below the critical strain rate. For the different amorphous alloys considered, this complexity can be reflected by the difference in the strain rate effect on the fracture angle [11,12,13,14,15,20] and the critical strain rate.

In the following, we try to explain the change of the plasticity at different strain rates. As we know, the plasticity of amorphous alloys is mainly contributed to the initiation and propagation of a large number of shear bands. The reconstruction in the shear band contributes to the repeated activation and arrest of the shear band, which leads to a decrease in the number of shear bands that can be generated during deformation as the strain rate increases, that is, the plasticity decreases as the strain rate increases. For the quasi-static cases, both the weakly thermal fluctuation and the enough time for reconstruction in the shear band, which eventually results in the relatively large plastic flow, as demonstrated in Figure 5. As for high-rate cases, the high loading rate allows almost no time for the structure recovery in the shear band. the initiation of the shear band under dynamic conditions is accompanied by a serious thermal softening, which will result in a significant decrease in the material′s viscosity in the shear band and then a reduction in the flow stress. Consequently, there is a fast propagation of the shear band until the finally catastrophic failure. This is consistent with the stress–strain curves presented in Figure 5, i.e., the stress initially reaches a maximum and then there is a decline, which is in accordance with the findings reported by other researchers in Zr-based BMGs [11,12,14,15,20]. These findings are significant for a proper understanding of the relationship between the strain rate and deformation behaviors, as well as a further development of the high mechanical performance for amorphous alloys.

## 4. Conclusions

For ZC3 amorphous alloy, the nominal yield strength exhibits a negative strain rate effect over a wide range of strain rates at room temperature. When the strain rate is less than 400 s^−1^, the nominal yield strength slowly decreases with an increase in the strain rate. When the strain rate is greater than 400 s^−1^, the yield strength decreases rapidly with increasing strain rate. However, the fracture angle increases with an increasing strain rate from 44° at 10^−5^ s^−1^ to 60° at 4000 s^−1^. Based on the experimental results of present study, we introduced two new parameters to describe the effects of the amorphous alloy composition and shear strain rate on the nominal yield strength to improve the CSM model. The model predicts that the normalized yield strength will linearly descend with logarithmic strain rate under quasi-static and medium strain rates. Because of the increase in the adiabatic temperature of the shear band under a high strain rate, the normalized yielding strength rapidly decreases linearly with the square of the strain rate. The model predictions and experimental results are in good agreement. These findings are significant for a proper understanding of the relationship between the strain rate and the yield strength and for the further developing the high mechanical performance of amorphous alloys.
